# The silent alarm: Ethical and cognitive discomfort in palliative care decision-making

**DOI:** 10.1017/S1478951526103149

**Published:** 2026-07-15

**Authors:** Júlia Drummond de Camargo, João Carlos Geber-Júnior

**Affiliations:** 1 Pallitive Care Team, Hospital Samaritano de Sao Paulo, São Paulo, Brazil; 2 Faculdade de Medicina Deparment, Universidade de São Paulo, São Paulo, Brazil; 3 Palliative Care Support Team, Hospital Samaritano Higienópolis, São Paulo, Brazil, Hospital Samaritano de São Paulo, São Paulo, Brazil

Palliative care unfolds at the intersection of clinical complexity and existential vulnerability, permeating different stages of the patient’s illness trajectory. As the disease progresses, discussions regarding advanced care planning and decision-making in various scenarios become increasingly urgent.

End-of-life decisions are complex processes involving multiple factors and are not always guided solely by technical protocols; practice teaches us that they are, above all, relational and cultural acts. When we ignore this dimension, we risk naturalizing our own professional perspective as the sole reference standard, thereby silencing the patient’s values and reducing autonomy to a merely procedural concept (Vidal et al. 2022; Geber-Junior 2026a).

## The blind spot at the bedside: biases under pressure

In clinical practice, as we gain experience, professionals begin to recognize that end-of-life decisions are more than technical choices. The variability of care can be related to different end-of-life care situations, as well as the diversity of individual values and preferences, and varying degrees of training and education regarding end-of-life care within the healthcare team (Siegel 2009; Moritz et al. 2011; Forte et al. 2018).

Decision-making is surrounded by scenarios of uncertainty, which favors the emergence of cognitive biases, such as confirmation, anchoring, and availability (Featherston et al. 2020). In the routine of high workload and team fragmentation, the mind seeks shortcuts. Biases like anchoring or confirmation are not merely theoretical concepts; they manifest when, under stress, we stop listening to the patient as a singular person and begin seeking only information that confirms our prior conduct, allowing communication to become defensive rather than relational (Geber-Junior and Forte 2025).

The danger lies in the invisibility of these processes: professionals believe that they are being objective while being led by “mental shortcuts” aimed only at reducing the discomfort of uncertainty. When this occurs, we fail to genuinely listen to the patient and instead naturalize our own cultural perspective as the only correct standard. At this moment, the practical reflection we must undertake is: Am I seeing this patient in their singularity, or am I merely reacting to another case that I experienced last week?

Recognizing that our objectivity is limited is the first step toward care that does not silence the values of those we serve.

## Cognitive dissonance as an ethical alarm

Frequently, we feel a deep cognitive discomfort – a “negative feeling” – when making a decision that contradicts our values of respecting autonomy, such as indicating an ICU bed against the patient’s prior wishes. We call this presence of discomfort, evidenced as a negative feeling caused by the contradiction between our beliefs and attitudes or by holding two inconsistent views on the same problem, cognitive dissonance, first described by social psychologist Leon Festinger in 1957 (Festinger 1957).

In clinical practice, this malaise is common in decisions that contradict living wills and the patient’s prior desires (Croskerry 2013). We must note that the extent to which this behavior manifests depends on the magnitude of the existing dissonance and the expectations surrounding the content; that is, the greater the magnitude of the dissonance, the greater the pressure to reduce it, leading us to seek an increasing number of justifications for our decisions (Harmon-Jones 2019).

Instead of silencing this perceived discomfort with rationalization or automatic technical justifications, we should attempt to become aware that we are experiencing cognitive dissonance and treat it as a warning sign that the patient’s autonomy may be at risk.

## The blind spot: perception vs. reality

Considered a scenario where professionals with an “obstinate” decision-making pattern often justify their conduct incoherently: they sincerely believe they value autonomy and share decisions, while their practical actions show the opposite (de Camargo and Forte 2024). This dissonance between attitudes and values we believe to be most appropriate forces us into humility and practical reflection: Is the care I deliver truly the care the patient desires or is it just the version my mind created to reduce my own discomfort?

Under the effect of stress – common in palliative care when facing difficult situations and communications – we are also susceptible to a considerable increase in our own biases, leading us to the unconscious use of mental shortcuts that seek to minimize the cognitive discomfort present in the dissonance between values and actions.

This discrepancy can be perceived in the associations between the frequency of cognitive biases and potentially inadequate clinical outcomes, where we risk taking decisions that are not aligned with the patient’s values and desires, motivated by our own fear of future regret (Tör-Çabuk and Koç 2024).

## Impact on clinical practice

Unresolved dissonance is not an individual problem; it contaminates the team’s relational environment and overflows into interpersonal relationships, potentially creating environments filled with resentment, burnout, and a deep sense of helplessness (Croskerry 2013; Ozdemir and Finkelstein 2018).

Silence in the face of dissonance disrupts the coherence of care and generates moral distress. When uncertainty is acknowledged with composure, it becomes open to discussion and ceases to be a source of defensive silence.

When pressure is high, the search for justifications to cognitively ground our decisions also increases (Cooper 2012; Hinojosa et al. 2016; Harmon-Jones 2019). The impact of cognitive dissonance at end-of-life decisions directly relates to the care provided when it conflicts with prior preferences, values, and even documented advance care planning (Rothgerber 2014).

Failure to recognize our biases and cognitive discomfort results in emotional disengagement and a decline in the quality of care provided (Croskerry 2013; Featherston et al. 2020). Therefore, investing in ethical education and psychological support must be considered an integral part of professional training and practice (Rothgerber 2014; Tör-Çabuk and Koç 2024).

## Conclusion: the ethics of epistemic humility

Recognizing our cognitive fallibility is not a weakness, but an ethical necessity. By accepting that we are subject to intuitive processes and automatic justifications, we create space for a truly shared practice, where technical decisions go hand-in-hand with the deepest values of those we care for.

To improve care, ethical education must go beyond theory and focus on metacognition – the act of thinking about one’s own thinking–transforming our team meetings into spaces for supervision where vulnerability is seen as a source of collective insight (Rivas et al. 2022).

True excellence in palliative care does not reside in the absence of doubt, but in the courage to sustain dialogue and re-evaluate decisions in the face of uncertainty, remaining ethically present at the limits of intervention rather than escaping discomfort through automatic action (Geber-Junior 2026b), opening space for care that is truly centered on the dignity and values of those we care for.

Being aware that we are cognitively fallible and that our judgments may be biased makes us professionals more capable of offering care that is, at once, technically sound and deeply human.
